# Xenografts in septic vascular surgery

**DOI:** 10.1007/s00772-016-0160-8

**Published:** 2016-07-12

**Authors:** I. Töpel, C. Uhl, I. Ayx, M. Steinbauer

**Affiliations:** 1 Klinik für Gefäßchirurgie, Krankenhaus Barmherzige Brüder Regensburg, Prüfeninger Str. 86, 93049 Regensburg, Germany; 2 Institut für diagnostische und interventionelle Radiologie, Neuroradiologie und Nuklearmedizin, Barmherzige Brüder Regensburg, Regenburg, Germany

**Keywords:** Infection, Vascular replacement, Xenogeneic materials, Bovine patch, Omniflow, Infektion, Gefäßersatz, Xenogene Materialien, Boviner Patch, Omniflow

## Abstract

**Background:**

In general, autologous veins are the optimal replacement material for an infected vascular graft in terms of handling, durability and resistance to reinfection. In the absence of suitable autologous material, several options are available, each of which has specific advantages and drawbacks with regard to these characteristics.

**Methods:**

In recent years, xenogeneic materials (in particular pericardial patches from different species and biosynthetic grafts) have been increasingly used as replacement material in the setting of infections. Bovine and equine pericardial patches are applied in particular as self-made tube grafts in the aortic region and also in infections of iliacofemoral prosthetic grafts and shunt infections.

**Results:**

The results of small clinical series on durability and resistance to reinfection are promising.

**Conclusion:**

It is feasible to use biosynthetic materials to replace infected intracavitary and extracavitary vascular grafts with remarkably low reinfection rates; however, the unique mechanical properties of the grafts as well as the initially increased thrombogenicity, need to be taken into consideration.

## Zusammenfassung

**Hintergrund:**

Die autologe Vene stellt im Allgemeinen das beste Ersatzmaterial für eine infizierte Gefäßprothese in Hinblick auf Handling, Langlebigkeit und Infektresistenz dar. Bei fehlendem autologem Material stehen verschiedene Optionen zur Verfügung, deren Anwendung bezogen auf diese Eigenschaften jeweils spezifische Vor- und Nachteile aufweist.

**Methoden:**

In den letzten Jahren werden vermehrt xenogene Materialien (insbesondere Perikard-Patches verschiedener Spezies und biosynthetische Prothesen) als Ersatzmaterial in der Infektsituation verwendet. Bovine und equine Patches kommen dabei insbesondere als selbstgefertigte „tube grafts“ im aortalen Bereich, aber auch bei Infektionen der iliakofemoralen Strombahn oder bei Shuntinfektionen zum Einsatz.

**Ergebnisse:**

Die Ergebnisse kleiner Serien sind in Bezug auf Langlebigkeit und Infektresistenz sehr vielversprechend.

**Schlussfolgerung:**

Die Anwendung biosynthetischer Prothesen als Rekonstruktionsmaterial bei intra- und extrakavitären Protheseninfekten ist möglich, insbesondere die Reinfektraten sind sehr niedrig. Allerdings müssen bei der Implantation die besonderen mechanischen Eigenschaften und die höhere initiale Thrombogenität der Prothesen beachtet werden.

Xenogeneic materials are increasingly being applied in arterial reconstruction procedures in vascular surgical routine. Their availability, good handling and high resistance to infection have led to these grafts also being used for procedures in the septic surgery environment. Initial results from application studies are now available.

Due to its high resistance to infection and excellent long-term stability, autologous vascular grafts nowadays represent the best method in septic vascular surgery; however, should this option be unavailable, alloplastic materials lag far behind despite various modifications (e.g. silver, Triclosan) to the vein in terms of the characteristics mentioned. Furthermore, the removal of (in particular deep) veins causes additional access morbidity and significantly prolongs procedural times. The use of biological graft materials was restricted for a long time due to the limited long-term function, degeneration and aneurysm formation, as well as high occlusion rates (particularly in peripheral areas). In view of the age and comorbidity-related reduction in life expectancy among patients requiring vascular surgery due to underlying infectious diseases or septic complications, allografts have come to play an important role in clinical routine, particularly in the case of intracavitary infections; however, since the revised version of the German Tissue Act (*Gewebegesetz*) came into effect in 2009, the procurement, processing, storage and transplantation of human tissue are subject to strict requirements, significantly limiting the availability of these materials. Xenogeneic grafts now hold a firm place in elective vascular surgical practice. The term (Greek ξένος, *xénos*, stranger) refers to materials of non-human biological origin. Originally, the shortage of suitable human donor organs led to attempts to transplant non-human organs but acute rejection processes posed the greatest obstacle. These mechanisms can now be even further suppressed in pigs as donor animals for human heart transplants by breeding knockout lines for superficial antigens and the use of immunoadsorption techniques. Despite this progress, xenotransplantation of living tissue or functional organs has not yet reached the clinical routine. Denatured xenogeneic vessels are not suitable for vascular replacement due to their tendency to degenerate and high thrombogenicity. Human Allo-Grafts used in vascular surgery are rendered cell-free and hence epitope-free, by means of special processing. Thus, they induce no specific immune response. This article discusses the role that the various currently available grafts can play in the setting of infection.

## Pericardial patches

The industry offers patches originating from a variety of species. Bovine and porcine pericardial patches are those most commonly used. They differ in terms of biomechanical properties, in particular material thickness and tensile strength. At our hospital, the bovine patch is used as the standard patch for elective procedures in all vascular areas excluding the popliteocrural region. The porcine patch is better suited for this region due to its low material thickness. Bovine pericardial patches have several advantages compared with alloplastic materials; however, these advantages are based purely on experience and have not yet been documented in an evidence-based manner. They are nevertheless characterized by good handling, high material strength and a low tendency to bleeding at the suture line. Their biocompatibility is significantly higher than that of alloplastic materials, a fact attributed to the collagenic fiber structure, which offers an ideal environment for the migration of fibroblasts and other receptor cells, thereby contributing to rapid integration and epithelialization [1]. Pericardial patches are available in a variety of sizes and are easy to store. Thus, the additional preparatory tasks required for the harvest of a venous graft are dispensed with. Bovine patch compliance is similar to that of an autologous artery, making it possible to achieve reconstruction that closely resembles the anatomical reality. This approach minimizes the compliance mismatch between wall and graft that contributes to restenosis due to intimal hyperplasia, particularly in endarterectomized segments. Finally, bovine tissue is a robust graft tissue without air inclusions, thereby permitting prompt ultrasound follow-up of the reconstructed area.

Infection rates also play an important role in the selection of vascular replacement materials. Compared with synthetic patches, only a small number of cases of patch infections have been published for bovine pericardium [2]. This factor, combined with the biophysical characteristics of this particular material, prompted a number of working groups to also use bovine patches in the setting of infected grafts. This applies not only to vascular surgery but also to other specialist surgical disciplines, such as thoracic surgery (reconstruction of the trachea and thoracic wall defects) [3] and cardiac surgery (in combination with valve repair procedures) [4].

The use of pericardial patches to replace the alloplastic patch infections seen relatively frequently in the inguinal region has already been described. McMillian et al. published a series of 51 patients in whom PTFE patches in the inguinal region were replaced with bovine pericardial patches [5]. Although methicillin-resistant *Staphylococcus aureus* (MRSA) was detected in as many as 22 % of the infections, 50 of the patches remained free of reinfection and did not require revision surgery over a mean follow-up of 2.1 years.

In the case of Szilagyi III inguinal infections repaired using bovine patches, the patch remains in place, unless bleeding is present (Zülke-Harnoss grade III) and treatment is carried out with debridement, sartorius myoplasty, negative pressure wound therapy (NPWT) and antibiotic therapy.

**Fig. 1:**
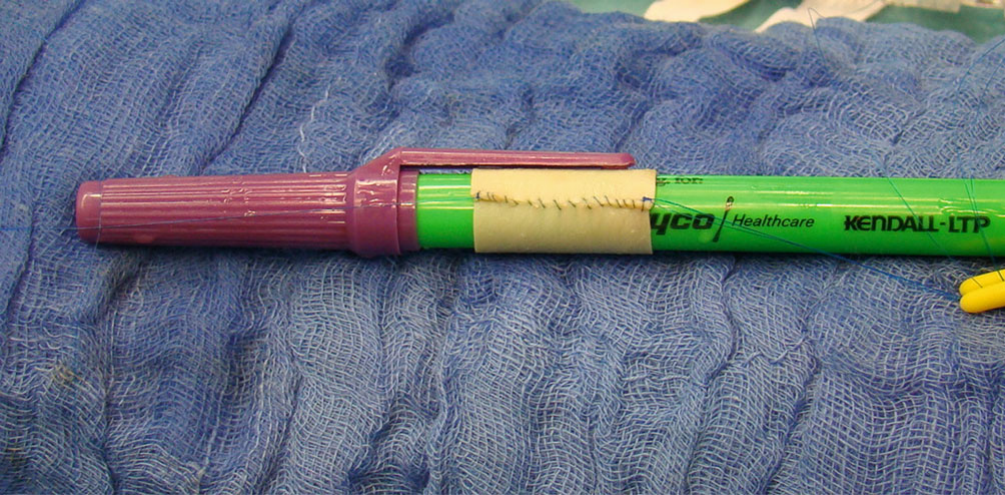
A tube is produced from a bovine patch over an 8 mm diameter cylinder. Non-absorbable sutures are used. In cases where it is not possible to accurately determine the required graft length, the suture is left uncompleted approximately 1–2 cm from the end of the patch to be completed in situ

Reconstruction using pericardial tubes has also been described in aortic graft infections and inflammatory changes in the native aorta. As early as 1997, the working group led by Pirelli [6] published five cases of infected infrarenal grafts that were replaced in situ using pericardial tubes [6]. In 2009 Motokawa et al. reported on the replacement of the thoracic aorta for an aortobronchial fistula [7] and the infrarenal aorta for a mycotic aneurysm [8] using a custom-made equine pericardial roll graft. Then, in 2011, Schmidlis’ group [9] published a series of 15 patients with graft infections following open or endovascular treatment, in whom grafts were removed and replaced with pericardial tubes. Of the patients 4 (27 %) died while still hospitalized due to the initial sepsis and multiorgan failure. No cases of reinfection occurred during the 2‑year follow-up period. Similarly, no graft degradation or aneurysms were observed [9]. Bürger and Gebauer [10] provided an excellent technical description of bovine pericardial patches as bypass material in the setting of infections. Large pericardial patches (10 ×15 cm or larger) are sutured to form a tube using non-absorbable monofilament suture material (Fig. 1). This can be performed free-hand in the case of large lumen grafts for aortic repair, while smaller lumen interposition grafts can be sutured over sterile cylinders of the appropriate diameter (e.g. chest drainage tubes and Hegar pins). Pericardial patches can be combined with other graft materials, such as biosynthetic grafts, veins or homografts [11].

**Fig. 2:**
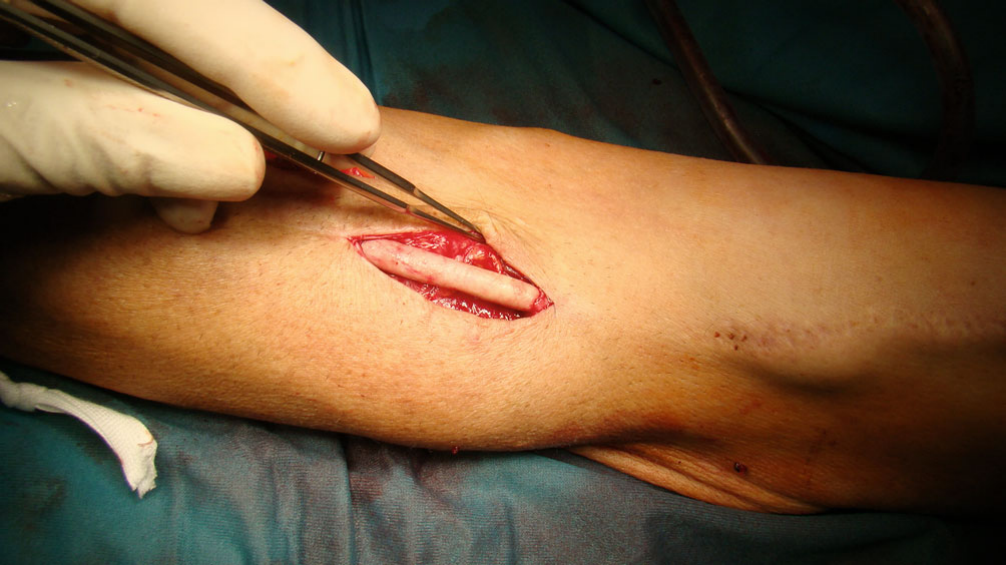
Partial replacement of a graft shunt in the upper arm with a bovine tube due to an infected aneurysm

We also use the bovine pericardial patch as graft material for localized infection of plastic prostheses in shunt surgery (Fig. 2). Here, it is important to place the suture lateral to the direction of puncture in order to avoid damage during shunt puncture and pseudoaneurysm formation. A healing time of 4–6 weeks should be allowed prior to initial puncture.

## Biosynthetic grafts

**Fig. 3:**
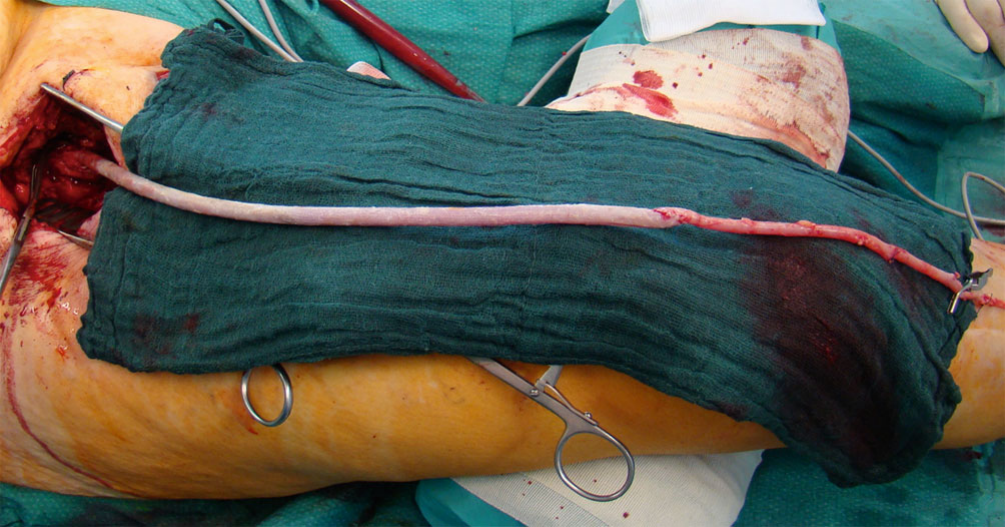
Omniflow composite bypass and vein to reconstruct an infected femorocrural bypass to the posterior tibial artery. The composite anastomosis is constructed as an oblique end to end anastomosis

Biosynthetic grafts consist of a polyester mesh enclosed in ovine collagen according to the principle of a Sparks mandril graft. Once processed, involving in particular fixation with glutaraldehyde, a stable collagen matrix forms. Due to the low infection rates when used in shunt surgery and elective bypass surgery as well as the encouraging results with xenogeneic patches in the infected surgical setting, efforts have also been made in recent years to evaluate biosynthetic grafts as graft materials for infections. Initial results on infected infrainguinal prosthetic grafts were published by our working group in 2012 [12] and by Fellmer et al. in 2014 [13]. These investigations showed that, in the absence of venous material, it is possible to replace infected inguinal grafts using Omniflow II® grafts and achieve goods results. No reinfections were observed in the mean follow-up period of 2 years. We recommend using the Omniflow II® prosthesis for femorocrural reconstruction due to its lower kink resistance; where possible, the use of composite grafts with autologous veins is preferred (Fig. 3). In addition, due to the initially higher thrombogenicity, more intensive anticoagulation and antiaggregation therapy is required, at least until the healing process is completed.

**Fig. 4:**
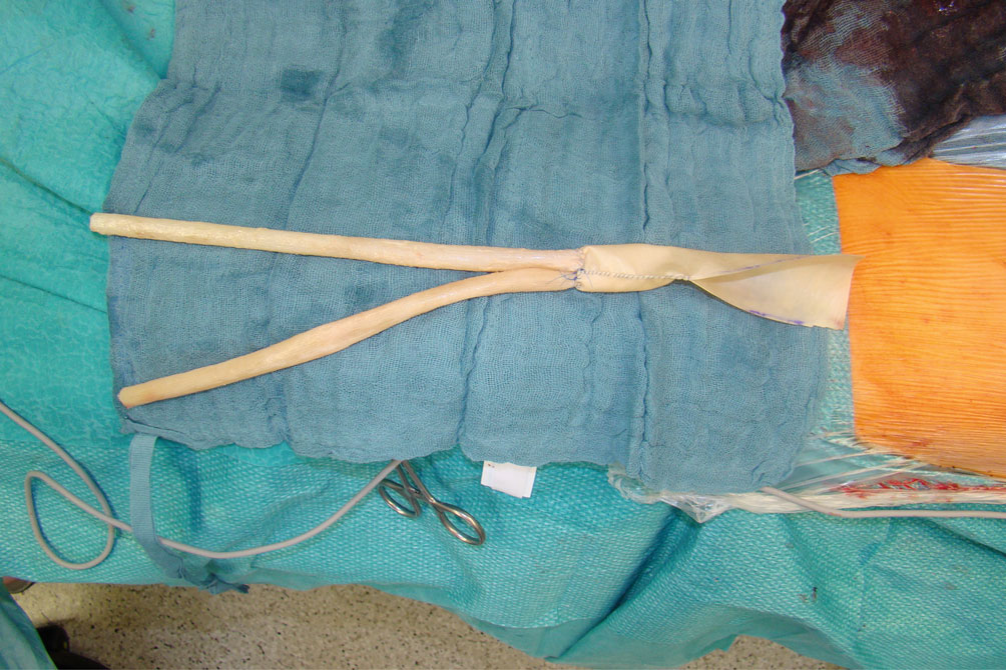
An aortobifemoral composite graft is produced using a pericardial tube and two Omniflow II grafts (8 mm diameter)

**Fig. 5:**
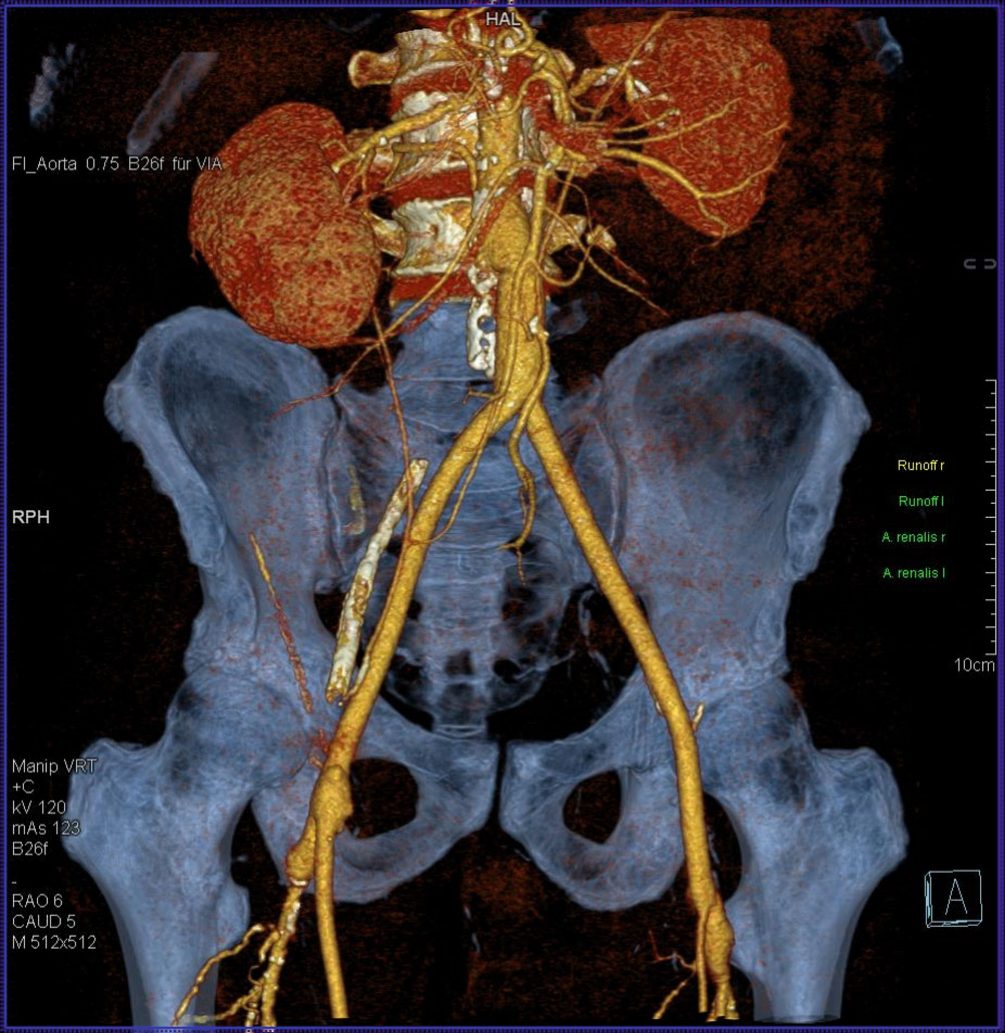
The graft appears normal on postoperative follow-up computed tomography

In December 2015 Krasznai et al. published a series of three patients with infected aortic grafts that could be reconstructed in situ using biosynthetic material [14]. All infections were resolved. We also use the Omniflow II® prosthesis as replacement material for infected aortobiiliac and aortobifemoral prosthetic grafts (Töpel et al. International Surgical Science [ISS] in press [to be published in 2016]), sometimes in combination with other materials (Fig. 4 and 5). There were no cases of reinfection among our patients, too.

## Summary

Xenogeneic grafts have found broad application in vascular surgery in the form of pericardial patches. There is a growing body of clinical data to suggest a rationale for their use in the contaminated surgical field or even as replacement material for infected synthetic patches and grafts in certain situations. Biosynthetic vascular grafts are highly resistant to infection when used in the elective setting; however, their use as replacement material for infected infrainguinal vascular grafts is only justified if autologous venous material is absent or insufficient and if good results have been seen in small application studies. Their special mechanical properties require particular consideration, most notably in femorocrural applications. Initial experience with their use in intracavitary graft infections has been gained.

## Conclusion

With careful preoperative evaluation and patient selection, xenogeneic grafts represent a valuable treatment option in septic vascular surgery. The use of biosynthetic grafts in particular is technically challenging and follow-up requires experience and careful attention.

## References

[1] Menger MD, Hammersen F, Messmer K (1992). In vivo assessment of neovascularization and incorporation of prosthetic vascular biografts. Thorac Cardiovasc Surg.

[2] Li X, Guo Y, Ziegler KR (2011). Current usage and future directions for the bovine pericardial patch. Ann Vasc Surg.

[3] Miller DL, Force SD, Pickens A, Fernandez FG, Luu T, Mansour KA (2013). Chest wall reconstruction using biomaterials. Ann Thorac Surg.

[4] Sündermann SH, Biefer HR, Emmert MY, Falk V (2012). Use of extracellular matrix materials in patients with endocarditis. Thorac Cardiovasc Surg.

[5] McMillan WD, Leville CD, Hile CN (2012). Bovine pericardial patch repair in infected fields. J Vasc Surg.

[6] Odero A, Argenteri A, Cugnasca M, Pirrelli S (1997). The crimped bovine pericardium bioprosthesis in graft infection: preliminary experience. Eur J Vasc Endovasc Surg.

[7] Yamamoto H, Yamamoto F, Ishibashi K, Chida Y, Minamiya Y, Nanjo H (2009). In situ replacement of the thoracic aorta using an equine pericardial roll graft for an aortobronchial fistula due to aortic rupture. Gen Thorac Cardiovasc Surg.

[8] Yamamoto H, Yamamoto F, Ishibashi K, Motokawa M (2009). In situ replacement with equine pericardial roll grafts for ruptured infected aneurysms of the abdominal aorta. J Vasc Surg.

[9] Czerny M, von Allmen R, Opfermann P (2011). Self-made pericardial tube graft: a new surgical concept for treatment of graft infections after thoracic and abdominal aortic procedures. Ann Thorac Surg.

[10] Bürger T, Gebauer T (2013). Boviner Perikardpatch als Bypassmaterial in der Infektsituation. Gefasschirurgie.

[11] Stöckl K, Steinbauer M (2015). Operative Therapie bei Infekt einer aortobifemoralen Y‑Prothese sowie Reinfekt eines femoropoplitealen (P1) Silberkunststoffbypasses links. Gefasschirurgie.

[12] Töpel I, Betz T, Uhl C, Wiesner M, Bröckner S, Steinbauer M (2012). Use of biosynthetic prosthesis (Omniflow II(R)) to replace infected infrainguinal prosthetic grafts-first results. Vasa.

[13] Fellmer PT, Wiltberger G, Tautenhahn HM, Matia I, Krenzien F, Jonas S (2014). Frühergebnisse nach peripherem Gefäßersatz mit einer biosynthetischen Kollagenprothese bei protheseninfektionen. Zentralbl Chir.

[14] Krasznai AG, Snoeijs MGJ, Siroen MP (2015). Treatment of aortic graft infection by in situ reconstruction with Omniflow II biosynthetic prosthesis. Vascular.

